# Clinical characteristics and outcomes of lung cancer patients with COVID-19: A systematic review and meta-analysis protocol

**DOI:** 10.1371/journal.pone.0273691

**Published:** 2022-08-31

**Authors:** Mingyue Wu, Siru Liu, Yi Yang, Jie Lin, Jialin Liu

**Affiliations:** 1 Information Center, West China Hospital, Sichuan University, Chengdu, China; 2 Department of Biomedical Informatics, Vanderbilt University Medical Center, Nashville, Tennessee, United States of America; 3 Department of Oral Implantology, West China Hospital of Stomatology, Sichuan University, Chengdu, China; 4 Department of Medical Informatics, West China Medical School, Sichuan University, Chengdu, China; Xiamen University - Malaysia Campus: Xiamen University - Malaysia, MALAYSIA

## Abstract

**Background:**

COVID-19 is spreading rapidly worldwide, and the population is generally susceptible to SARS-CoV-2, especially those with cancer. Hence, our study aims to design a protocol for a systematic review and meta-analysis of the clinical characteristics and prognoses of lung cancer patients with COVID-19.

**Methods:**

The protocol is prepared following the preferred reporting items for systematic reviews and meta-analyses (PRISMA) guidelines. The literature will be searched in Embase, Pubmed, the Cochrane Library, LitCovid, and CNKI for potentially eligible articles. The quality of the articles will be used in the Newcastle-Ottawa Quality Assessment Scale (NOS) and Cochrane Handbook for Systematic Reviews of Interventions. Statistical analysis will be performed through RevMan 5 software. This review protocol has been registered in PROSPERO (CRD42022306866).

**Discussion:**

To clarify whether COVID-19 affects the clinical symptoms and prognoses of lung cancer patients. Further study is needed to establish the best evidence-based for the management of lung cancer patients with COVID-19.

**Conclusion:**

The definitive conclusion will be important to physicians effectively manage lung cancer patients with COVID-19.

## Introduction

The COVID-19 epidemic continues to spread all over the world. The novel highly transmissible Omicron variant of COVID-19 may become the dominant strain of the virus. The clinical symptoms show new features and the number of infections patients continue to rise. As of February 6, 2022, more than 392 million confirmed cases and more than 5.7 million deaths have been reported worldwide, with a patient mortality rate of 1.44% [1]. COVID-19 is generally susceptible to the population, especially in cancer patients [2]. This may be related to the weakened immunity of cancer patients [3]. Some studies have shown that the proportion and number of lymphocytes, especially the number of T-lymphocytes, are reduced to varying degrees in COVID-19 patients [4]. The infection rate and severity of COVID-19 are closely related to the immune function of the patient [5, 6]. At the same time, when a malignant tumour appears in the organism, the factors secreted by tumour cells can reduce the level of lymphocytes and lead to impaired immune response [7]. The decreased ability of the organism to recognize and kill mutated cells not only accelerates the growth of tumour cells but also makes cancer patients vulnerable to COVID-19 infection. It has been shown that cancer patients have a higher risk and worse prognosis for COVID-19 infection compared to non-cancer patients.

**Fig 1:**
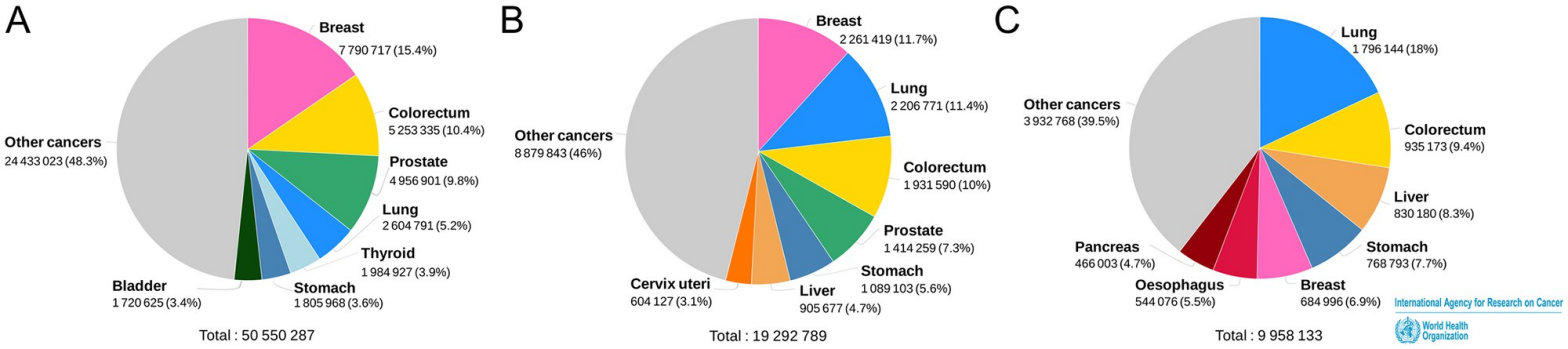
The worldwide estimated number of cancer in 2020. (A) Estimated number of prevalent cases (5-year) in 2020, worldwide, both sexes, all ages. (B) Estimated number of new cases in 2020, worldwide, both sexes, all ages. (C) Estimated number of deaths in 2020, worldwide, both sexes, all ages. (Datasource: Globocan2020 Graph production: Global Cancer Observatory).

According to the latest report of the World Health Organization (WHO), there were 50.55 million prevalent cases (5 years) of cancer, including 2.60 million lung cancer patients (5.2%) in 2020. And there were an estimated 19.3 million new cancer cases (18.1 million, excluding non-melanoma skin cancer) and nearly 10 million cancer deaths (9.9 million, excluding non-melanoma skin cancer) in 2020 [8]. Lung cancer was an estimated 2.2 million new cases in 2020, with an estimated 1.8 million deaths (18%), making it the second most common cancer in the world (Fig 1) [9]. Therefore, the prevention and treatment of lung cancer with COVID-19 is essential. Several studies have reported that lung disease and cancer are risk factors for mortality outcomes in patients with COVID-19 [2, 10–18]. However, the evidence is still inadequate. This study will evaluate the clinical characteristics and prognoses of lung cancer with COVID-19 through a systematic review and meta-analysis, and provide an evidence-based basis for the management of lung cancer with COVID-19.

## Methods

### Study design

We completed the systematic review protocol following Preferred Reporting Items for Systematic Reviews and Meta-Analyses (PRISMA) [19]. Furthermore, this systematic review protocol has been registered at the International Prospective Registry of Systematic Reviews (PROSPERO) (registration number: CRD42022306866). The study will use published data and does not require ethical approval.

### Eligibility criteria

The inclusion and exclusion criteria are based on the construction of systematic reviews with the PICOS. Each aspect is clearly defined as follows (Table 1).

**Table 1:** PICOS of the study.

Construction	Contents
Participants (P)	Patients were diagnosed with lung cancer and COVID-19 by laboratory or pathology, regardless of age, gender, and order of diagnosis.
Intervention (I)	Not applicable.
Comparison (C)	Lung cancer patients without COVID-19 served as the control group.
Outcomes (O)	Pertinent clinical characteristics included fever, cough, dyspnea, imaging manifestations, laboratory indicators, and pathological findings. Outcomes data, including mortality, ICU hospitalization rate, intubation rate, disease outcome, adverse events, and follow-up, were also extracted.
Study (S)	All observational research, case report, cohort study, and prospective studies were included in the study. The abstracts, editorials, review articles, commentaries, and guidelines are excluded. All included papers were published from November 1, 2019 to June 1, 2022.

### Search strategy

The literature search process will be performed independently by two researchers, including the preservation of online searches, deduplication and screening of titles, abstracts and full texts. Possible disagreements will be resolved by consulting a third author.

We will search Embase, PubMed, Cochrane Library, LitCovid, and China National Knowledge Infrastructure (CNKI) databases for articles published in English and Chinese. A combination of Medical Subject Headings (MeSH) and textual data will be applied as the search strategy. MeSH terms used for our searches are "COVID-19", "sars-cov-2", "coronavirus disease 2019", "cancer", "Neoplasms", "carcinoma", "Lung", "Pulmonary", "outcome", "signs and symptoms" (Table 2). The search terms will be translated into Chinese when searching the Chinese database (CNKI).

**Table 2:** Details of the search strategy for Embase.

Steps	Search strategy
#1	’COVID-19’:ab,ti
#2	’sars-cov-2’:ab,ti
#3	’coronavirus disease 2019’:ab,ti
#4	#1 OR #2 OR #3
#5	’cancer’:ab,ti
#6	’Neoplasms’:ab,ti
#7	’carcinoma’:ab,ti
#8	’Lung’:ab,ti
#9	’Pulmonary’:ab,ti
#10	#5 OR #6 OR #7 OR #8 OR #9
#11	’outcome’:ab,ti
#12	’signs and symptoms’:ab,ti
#13	#11 OR #12
#14	#4 AND #10 AND #13

### Screening procedure

**Fig 2:**
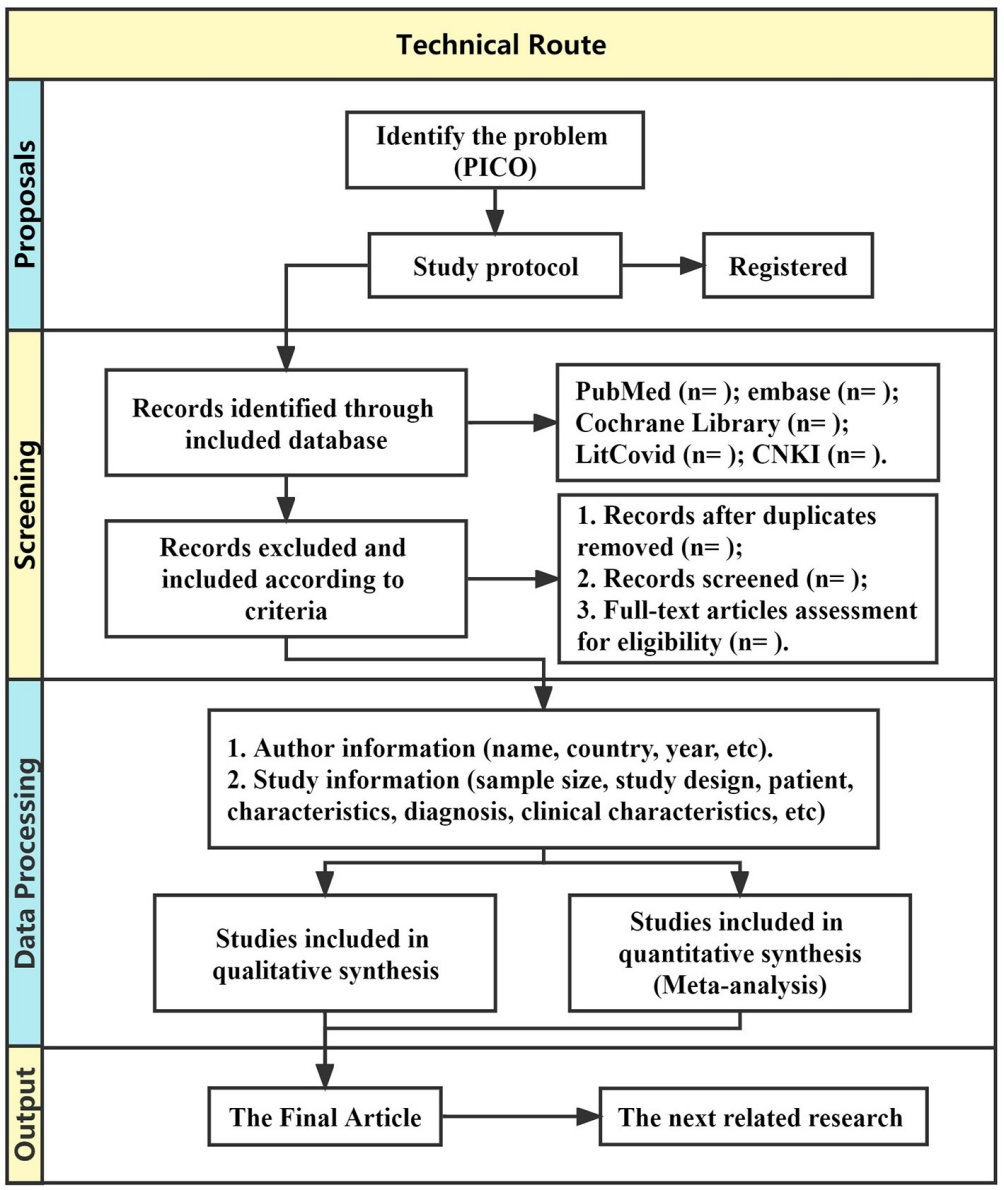
Flow chart of the study selection.

**Fig 3:**
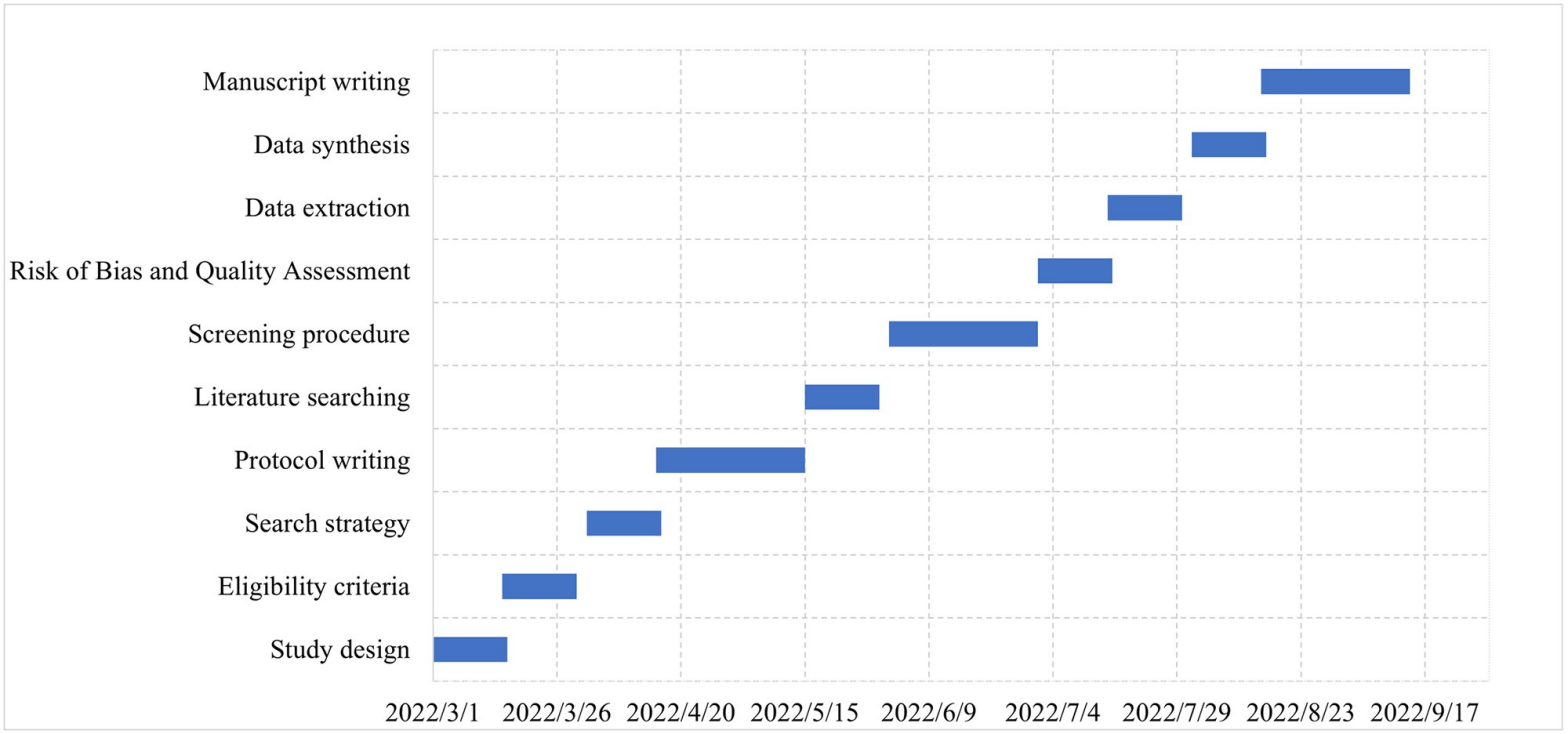
The Gantt chart of the status and timeline of the study.

For the visual display of a large amount of literature, the retrieved studies will be imported into RevMan 5 to remove duplicates. At first, two researchers (MW and SL) will independently screen the literature according to the titles and abstracts. Then, the full-text screening will be performed. Finally, two researchers will cross-check the included studies, disagreements will be resolved by discussion between the researchers until consensus is achieved. If disagreements persist, a third reviewer (JL) will make the final decision. The flow chart of the literature screening is shown in Fig 2. The Gantt chart of the status and timeline of the study is shown in Fig 3.

### Risk of bias and quality assessment

The Newcastle-Ottawa Quality Assessment Scale (NOS) will be used to assess the quality of case-control and cohort studies [20], and the Cochrane Handbook for Systematic Reviews of Interventions to assess randomized controlled trials (RCTs) [21]. Four authors will independently assess the quality and risk of bias of the included studies and cross-check them. Disagreements will be resolved through group discussion and mutual consultation.

### Data extraction

Two researchers (MW and SL) will independently extract these data and fill in the pre-set form. The extracted information was as follows:

1. Author information: name of the first author, country of the corresponding author, year of publication, name of the journal.
2. Study information: sample size, type of study design [(retrospective, prospective), (RCT, case-control, cohort study)], time range, patient characteristics, diagnosis, clinical characteristics (fever, cough, dyspnea, imaging manifestations, laboratory indicators, pathological findings), prognosis (mortality, ICU hospitalization rate, intubation rate, disease outcome), adverse events, follow-up, etc.

Two researchers will cross-check the extracted data. If there is disagreement, it will be discussed. If no agreement is reached, a third investigator (JL) will make the final decision. The study authors will be contacted for more information if needed.

### Data synthesis

The RevMan 5 software will be used for statistical analyses. The odds ratio (OR) or risk ratio (RR) and the 95% confidence interval (CI) will be used to estimate the dichotomous variables. The standard mean difference (SMD) and 95% CI will be used to describe continuous variables. The I^2^ test will be applied to assess the heterogeneity among the included studies. If the level of heterogeneity is not significant (P > 0.1, I^2^ < 50%), we will use the fixed-effect model; If there is heterogeneity among the studies (P ≤ 0.1, I^2^ ≥ 50%), we will use the random-effects model [22]. When heterogeneity is fairly high, subgroup analyses or meta-regression will be conducted to assess the possible sources of heterogeneity. Sensitivity analyses will be used to assess the stability of the results. We will use funnel plots, Egger’s test and Begg’s test to assess the publications bias [23].

### Ethics and dissemination

This is a systematic review and meta-analysis, so ethical approval is not required. The results of the systematic literature review and meta-analysis will be published in a peer-reviewed journal.

### The status and timeline of the study

This systematic review and meta-analysis is ongoing, and we estimate that it will be completed and reported within 12 months.

## Discussion

COVID-19 emerged as a health emergency in the 21st century across the globe. Despite the hope that the widespread vaccine use will reduce morbidity and mortality associated with COVID-19, the pathogenic and ever-mutating SARS-CoV-2 remains a major global concern [24]. While many infected individuals were reported to suffer pathological changes in multiple organs and systems such as lung, heart, liver, kidney, and gastrointestinal mucosa, the first organ with pathological changes was the lung, such as lung consolidation, infiltration of inflammatory cytokines, etc. [25–28].

Individuals with cancers are more susceptible to respiratory viruses due to systemic immunosuppressive states and overall poor health status caused by both cancer and anticancer treatments [29–32]. This has spawned worries that cancer patients are particularly vulnerable to severe COVID-19 infection. So far, several studies have evaluated the impact of cancers on the natural history and prognosis of COVID-19 [33–35]. Prior studies have shown that patients with active cancer or a history of malignant tumours suffer worse prognoses and outcomes [36–40]. Additionally, patients with cancers have an increased risk of mechanical ventilation rate and mortality compared with those individuals without cancers [41]. Liang et al. reported that the mortality and intensive care unit (ICU) admission rates of COVID-19 cancer patients were significantly higher than those of non-cancer patients. However, they ignored the effect of confounding factors, such as age, sex, and comorbidities, which were recognized to contribute to a worse prognosis [3, 38]. Recent research data demonstrate that the incidence rate and mortality of hospitalized COVID-19 cancer patients are similar to those of non-cancer COVID-19 patients after eliminating those influencing factors such as age and number of comorbidity [42].

Although the disease course varies, both COVID-19 and lung cancer begin in the lungs. Here comes a question, how will the combined effect of COVID-19 and lung cancer affect the clinical symptoms and prognosis of patients? Based on this problem, we conducted a preliminary search of the existing literature on COVID-19 and cancer, and we found most of the systematic reviews focus on the comparison of cancer and non-cancer patients [43–46]. However, there is no systematic review of lung cancer patients with and without COVID-19. Therefore, this review and meta-analysis will systematically explore the evidence available on lung cancer patients with COVID-19, and identify the risk factors or determinants associated with lung cancer. By collecting and summarizing information, it is possible to better understand how lung cancer and COVID-19 and related factors interact with each other, especially in the context of long-term COVID. However, with the new long-term COVID public health problem, there is an urgent need for evidence-based data to help manage lung cancer patients with COVID-19. This review will provide direction for further research.

However, this systematic review has some limitations. Our study included only peer-reviewed English and Chinese articles published in academic journals, which may bias the interpretation of the results. In addition, among the included studies, there may be differences in patient characteristics, severity, and complications between studies, which may also lead to clinical heterogeneity.

## Conclusion

The results of this study could provide physicians, patients and policymakers with the best clinical evidence in the management of lung cancer patients with COVID-19 patients. We hope that our findings will help fill the knowledge gap in this field and facilitate potential new research on lung cancer patients with COVID-19.

## Supporting information

S1 Checklist PRISMA-P 2015 checklist. (DOC)
